# Simulation and Validation in Brain Image Analysis

**DOI:** 10.1155/2016/1041058

**Published:** 2016-06-28

**Authors:** Jussi Tohka, Pierre Bellec, Christophe Grova, Anthonin Reilhac

**Affiliations:** ^1^Department of Bioengineering and Aerospace Engineering, Universidad Carlos III de Madrid, Avenida de la Universidad 30, 28911 Leganes, Spain; ^2^Instituto de Investigacion Sanitaria Gregorio Marãnon, Calle de Doctor Esquerdo 46, 28007 Madrid, Spain; ^3^Unité de Neuroimagerie Fonctionnelle, Centre de Recherche de l'Institut Universitaire de Gériatrie de Montréal, Université de Montréal, Montreal, QC, Canada H3W 1W5; ^4^Department of Computer Science and Operations Research, University of Montreal, Montreal, QC, Canada H3C 3J7; ^5^Physics Department and PERFORM Centre, Concordia University, 7141 Sherbrooke Street West, Montreal, QC, Canada H4B 1R6; ^6^Multimodal Functional Imaging Lab, Biomedical Engineering Department, McGill University, 3775 University Street, Montreal, QC, Canada; ^7^CERMEP-Imagerie du Vivant, PET-MR Department, Boulevard Pinel, 69677 Bron, France

The development of novel data analysis methods in brain imaging has been extremely fast paced in recent years. Advanced analytical tools are seen today as instrumental to further our understanding of the brain in health and disease. Translation to practitioners has also been accelerating, with the release of free and open-source software implementations of new tools starting to become the norm. Novel methods in brain imaging can often be used to ask new and exciting questions, and we are concerned that this may have relegated their validation to a position of secondary importance. Consequently, we believe that brain imaging as a field needs to improve its standards for method validation and that validation in itself is a research area where new methodologies are very much needed. Better validation steps could dramatically improve brain imaging methods in the future, by exposing the strengths and limitations of competing methods under a variety of experimental conditions and image acquisition protocols. These progresses will lead to more efficient and reproducible science and help equip brain scientists with the arsenal of high-quality tools they need for big data analytics.

The major challenge faced by researchers when validating brain imaging methods is the lack of a ground truth measure against which the outcome of their methods are compared. Unlike fields such as machine learning in natural images, there are only few public benchmark brain imaging datasets that get widely included in validation studies within a specific subfield. This lack of benchmarks makes it very difficult to compare validation results published on different image analysis methods. Developing reference benchmarks is very challenging because the physiological (e.g., neuronal and metabolic) and physical (e.g., magnetic and electrical) processes underlying brain imaging are extremely complex. Therefore, incorporating experimental imaging data into method validation is highly challenging, perhaps besides valuable and often expensive reproducibility studies. This challenge can be sometimes overcome in specific applications, for example, in image segmentation, when an automated method is expected to replace the manual work by a human expert. Yet, generating manual segmentation requires countless hours of work. Relying on pure simulations, on the other hand, is also challenging because of the aforementioned complexity of the physiology and data generating process. Simulations relying on a simple linear mixture of ground-truth signals and white noise can only be seen as a little more than a sanity check. Sound validation using simulated images needs to encompass detailed biological models as well as the bias inherent to employed imaging technique(s).

This special issue tries to fill some of the gap that exists between the analysis method development and the method validation. We received 14 papers out of which 5 were selected for the inclusion to the special issue. The accepted papers described validation techniques and their applications for multiple applications using different imaging techniques.

In “MRBrainS Challenge: Online Evaluation Framework for Brain Image Segmentation in 3T MRI Scans” A. M. Mendrik et al. describe an online platform to evaluate tissue segmentation in structural brain magnetic resonance imaging (MRI). They also report the evaluation results of MRBrainS13 competition for brain tissue segmentation of the aging brain. Manual segmentation is used as ground-truth to evaluate automatic segmentation algorithms.

In “Evaluation of Second-Level Inference in fMRI Analysis,” S. P. Roels et al. compare different modeling and inference techniques for general linear model (GLM) analysis of group functional magnetic resonance imaging. Although the GLM has been ubiquitous in fMRI research for decades, distinct variants remain popular to this date, and the validity of their underlying assumptions is not yet well established. How these variants behave in terms of reproducibility of results, instead of sensitivity and specificity, is also incompletely understood. S. P. Roels et al. implemented mixed effect simulations as well as a bootstrap analysis on real data to shed light on these questions.

In “How Many Is Enough? Effect of Sample Size in Inter-Subject Correlation Analysis of fMRI” J. Pajula and J. Tohka evaluate intersubject correlation (ISC) based analysis of fMRI data with a large dataset consisting of 130 subjects. They utilize split-half resampling to disclose the reproducibility of the analysis results with different sample sizes and additionally compare the analysis results using a large 130-subject dataset to the analysis results with smaller sample sizes.

In “MEG Connectivity and Power Detections with Minimum Norm Estimates Require Different Regularization Parameters” A.-S. Hincapie et al. evaluate the tuning of regularization hyperparameter within the minimum norm source reconstruction context when applied to magnetoencephalography (MEG) data. They addressed the question using Monte Carlo simulations of MEG data, where they generated 21,600 configurations of pairs of coupled sources with varying sizes, signal-to-noise ratio (SNR), and coupling strengths, thus providing a detailed evaluation framework within a new application context, the analysis of resting state functional connectivity using MEG data.

In “BrainK for Structural Image Processing: Creating Electrical Models of the Human Head,” K. Li et al. present BrainK, which is a set of automated procedures for characterizing the tissues of the human head from magnetic resonance images. The tissue segmentation and cortical surface extraction support the primary goal of modeling the propagation of electrical currents through head tissues. They compare the accuracies of BrainK's tissue segmentation and cortical surface extraction in relation to existing research tools (FreeSurfer, FSL, SPM, and BrainVisa). Their method is presenting and evaluating a very promising realistic head model, using finite element model, for electroencephalography (EEG) forward model. This is a necessary step to allow accurate source reconstruction (inverse problem) from high density EEG data.

